# *Prototheca zopfii* isolated from bovine mastitis induced oxidative stress and apoptosis in bovine mammary epithelial cells

**DOI:** 10.18632/oncotarget.16653

**Published:** 2017-03-29

**Authors:** Muhammad Shahid, Jian Gao, Yanan Zhou, Gang Liu, Tariq Ali, Youtian Deng, Naveed Sabir, Jingliang Su, Bo Han

**Affiliations:** ^1^ College of Veterinary Medicine, China Agricultural University, Beijing 100193, P R China

**Keywords:** bovine mastitis, Prototheca zopfii, bMECs, apoptosis, oxidative stress

## Abstract

Bovine protothecal mastitis results in considerable economic losses worldwide. However, *Prototheca zopfii* induced morphological alterations and oxidative stress in bovine mammary epithelial cells (bMECs) is not comprehensively studied yet. Therefore, the aim of this current study was to investigate the *P. zopfii* induced pathomorphological changes, oxidative stress and apoptosis in bMECs. Oxidative stress was assessed by evaluating catalase (CAT), superoxide dismutase (SOD), glutathione peroxidase (GPx), malondialdehyde (MDA) contents and lactate dehydrogenase (LDH) activity, while ROS generation and apoptosis was measured by confocal laser scanning microscopy. The results revealed that infection of *P. zopfii* genotype II (GTII) significantly changed bMECs morphology, increased apoptotic rate and MDA contents at 12 h (*p* < 0.05) and 24 h (*p* < 0.01) in comparison with control group, in time-dependent manner. LDH activity and ROS generation was also increased (*p* < 0.01) at 12 h and 24 h. However, SOD and CAT contents in bMECs infected with GTII were decreased (*p* < 0.05) at 12 h, while GPx (*p* < 0.01), SOD (*p* < 0.05) and CAT (*p* < 0.01) levels were reduced at 24 h. In case of GTI, only CAT and GPx activities were significantly decreased when the duration prolonged to 24 h but lesser than GTII. This suggested that GTII has more devastating pathogenic effects in bMECs, and the findings of this study concluded that GTII induced apoptosis and oxidative stress in bMECs via the imbalance of oxidant and antioxidant defenses as well as the production of intracellular ROS.

## INTRODUCTION

Bovine mastitis is an inflammatory response of the udder, which is characterized by pathological alterations in mammary gland and physio-chemical changes in milk. Bovine protothecal mastitis is mostly caused by *P. zopfii* [1]. *Prototheca* is achlorophyllous unicellular algae, which is closely related to chlorella species [2]. The cell wall of these microalgae contains sporopollenin, a robust biopolymer which makes the organism highly resistant to enzymatic degradation, mechanical injuries, physical and chemical treatments [3].

To date, six *Prototheca* species have been reported: *P. blaschkeae, P. cutis, P. stagnora, P. wickerhamii, P. ulmea*, and *P. zopfii* [2, 4]. *P. zopfii*, *P. cutis*, *P. wickerhamii* and *P. blaschkeae*are are causative agents of the protothecal infection in animals and human beings [4–6]. There are different clinical manifestations of protothecosis in different host species. *P. wickerhamii* and *P. zopfii* can cause protothecosis in human beings, characterized by local cutaneous lesions, olecranon bursal infection and also disseminated infection [7].

*P. zopfii* genotype-II (GTII) is linked with the most severe forms of protothecosis in animals, like bovine mastitis or canine encephalitis. Whereas genotype-I (GTI) is reported to be non-pathogenic in cattle [8–10]. Protothecal mastitis in cattle is characterized by abrupt decrease in milk production and increase in somatic cell count, which may lead to culling of dairy cow [11]. It is becoming serious problem for dairy cattle throughout the world due to its inherent resistance against different antimicrobials [12]. Various predisposing factors can exacerbate protothecal mastitis, like poor dairy herd management and unhygienic status, as under suitable conditions it can multiply in the surrounding environments as saprophytic form [13].

In bovine mastitis, oxidative stress occurs due to infection of pathogen that triggers the immune response and induces the reactive oxygen species (ROS) generation in cells [14]. The proliferation and apoptosis of bovine mammary epithelial cells (bMECs) under oxidative stress are modulated by cellular antioxidant status [15]. Lipid peroxidation in cell is due to the oxidative degradation of lipid which results in cell damage. Malondialdehyde (MDA) is a common indicator to assess the extent of lipid peroxidation [16]. The antioxidant defense mechanism of mammalian cells has the ability to counteract the detrimental effects of free radicals induced lipid peroxidation. Antioxidant enzymes such as catalase (CAT), superoxide dismutase (SOD) and glutathione peroxidase (GPx) react synergistically with each other to detoxify the lethal effects of lipid peroxidation [17]. Oxidative stress could contribute to compromise immunity and increase the severity of disease outcome, and also take part in the pathogenesis of bovine mastitis [18, 19]. The cytotoxic effect of oxidant is well known through protein, amino acid oxidation and DNA damage, as well as the ROS can take part in intracellular signals transmission. ROS reduces the antioxidant protection mechanisms and causes tissue damage in inflammatory process. Oxidative stress to bovine mammary secretary cells was proposed to decrease the secretary cells number and milk production [20]. In addition, lactate dehydrogenase (LDH) release assay could be used to evaluate cytopathic effect of pathogen [21]. LDH is normally present in the cytoplasm and release into the cell culture medium through damaged cell membrane. The bMECs are used as an alternative to *in vivo* study [22]. BMECs are less expensive and could be an ideal model to evaluate *in vitro* oxidative stress induced by *P. zopfii*, which may provide some comprehension in the pathogenesis of protothecal mastitis. Thus, in current study the activities of these enzymes were assessed to evaluate the oxidative stress in bMECs induced by *P. zopfii*.

The development of bovine protothecosis and the pathogenic diversity among the various *Prototheca* spp. and genotypes are scantily understood. Our earlier report was an attempt to study the immune response in bMECs challenged with *P. zopfii* remain inconclusive due to the fact that oxidative stress in bMECs induced by *Prototheca* infection was not investigated [23]. Therefore, the present study was conducted to investigate the effect of oxidant and antioxidant enzymes in bMECs after *in vitro* infection of GTI and GTII. Additionally, patho-morphological and apoptotic effects of both strains were also carried out by hematoxylin and eosin staining, and annexin V-FITC and PI staining, respectively.

## RESULTS

### *Prototheca zopfii* induced apoptosis in bMECs

**Figure 1:**
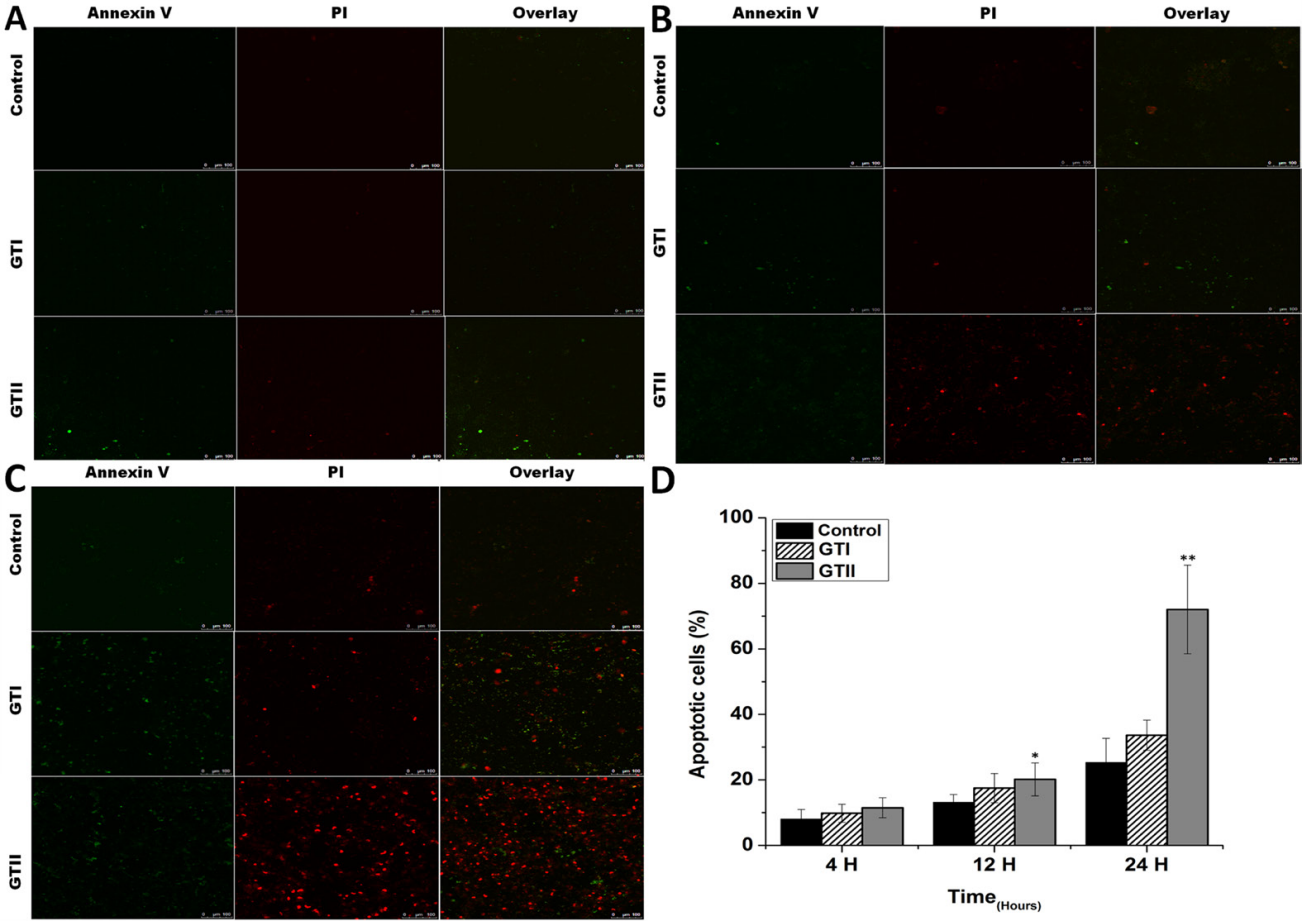
The bovine mammary epithelial cells stained with FITC-conjugated annexin-V (Green) and PI (Red) at 4 h (A), 12 h (B) and 24 h (C) after infection of *P. zopfii* GTI and GTII (**A**–**C**) The stained bMECs were observed by laser scanning confocal microscope, the upper panel represents normal cells, the middle panel shows representative bMECs treated with GTI, and the lower panel shows bMECs treated with GTII. (**D**) Shows the effects of *P. zopfii* GTI and GTII in bMECs apoptosis. *p < 0.05 and **p < 0.01 indicate the significance and highly significance differences.

To investigate the pathogenic effect of *P. zopfii* GTI and GTII on bMECs, the cultured bMECs were treated with *P. zopfii* GTI and GTII for 4 h, 12 h and 24 h using annexin V-FITC and PI staining. The apoptotic cells were significantly increased in case of *P. zopfii* GTII infected group both in early stage (annexin V-positive cells) and late stage (annexin V- and PI-double positive cells) on 12 h and 24 h (Figure 1A–1C). The number of apoptotic cells were increased from 20.16% ± 5.02% (*p* < 0.05) to 72.00% ± 13.5% (*p* < 0.01) with increase in time of infection from 12 h to 24 h (Figure 1D). However, the GTI could not induce significant apoptotic effect in bMECs as compared to control.

### Morphological alterations in infected bMECs

**Figure 2:**
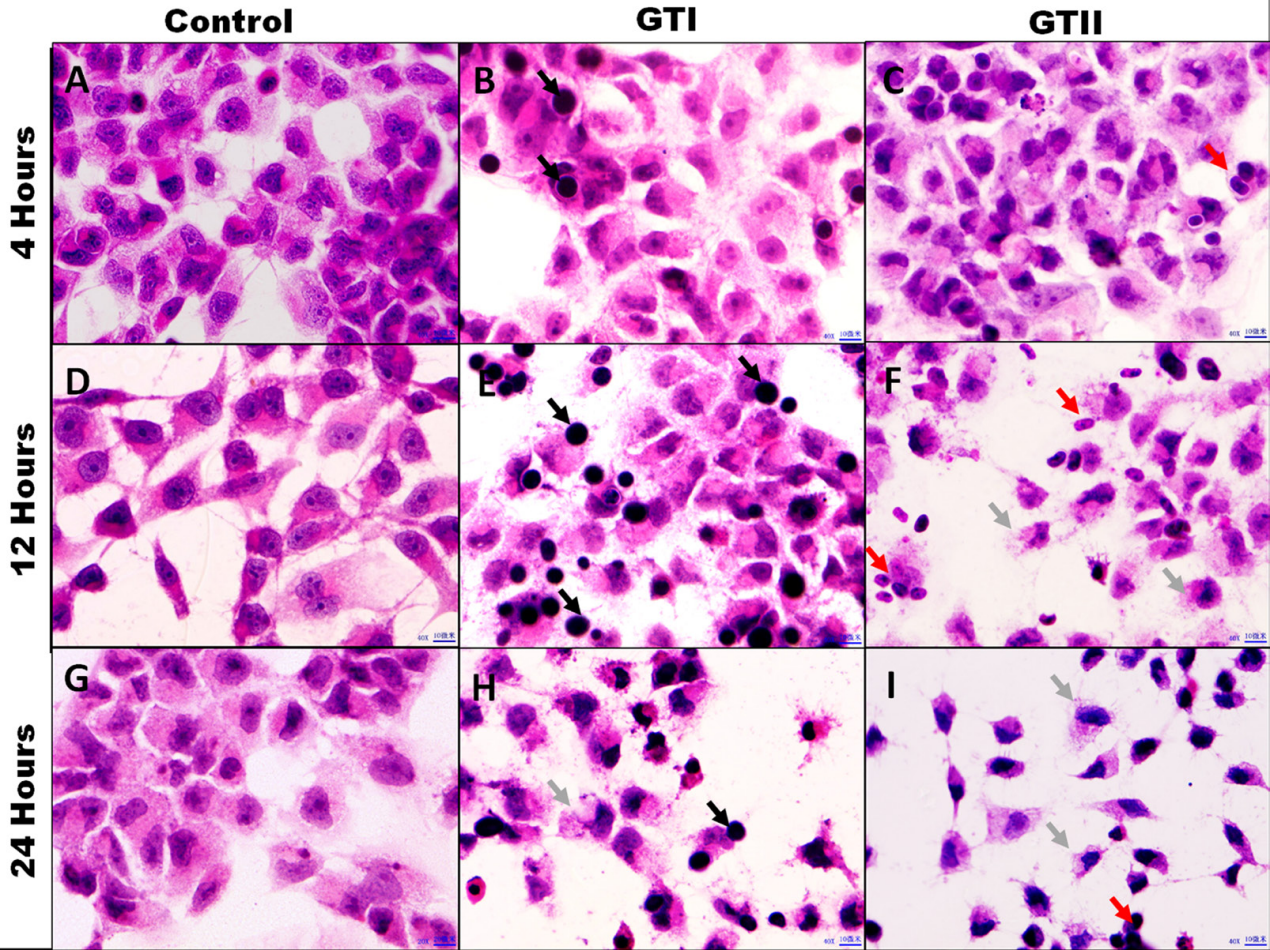
Morphological assessment of bMECs after infected with *P. zopfii* GTI and GTII Optical microscopy analysis. (**A**, **D**, **G**) Control 4 h, 12 h and 24 h; (**B**, **E**, **H**) *P. zopfii* GTI treatment for 4 h, 12 h and 24 h; (**C**, **F**, **I**) *P. zopfii* GTII treatment for 4 h, 12 h and 24 h. Black arrow shows adhesion of *P. zopfii* GTI with cells. Red arrow indicates adhesion of *P. zopfii* GTII with cells. Gray arrow demonstrates the cells patho-morphological changes.

The GTII infected bMECs exhibited discrete morphological changes evidenced by the loss of cellular limits and alterations in cell volume at 12 h and 24 h post-infection which showed the cytopathic effects of *P. zopfii* GTII (Figure 2F, 2I). While GTI depicted slight morphological changes at 24 h (Figure 2H). importantly, adhesion of both *P. zopfii* genotypes to bMECs was seen at 4 h (Figure 2B, 2C), 12 h (Figure 2E, 2F) and 24 h (Figure 2H, 2I) post-infection. Control group cells did not show any apparent alterations in cell morphology (Figure 2A, 2D and 2G).

### Oxidative stress indicators

**Figure 3:**
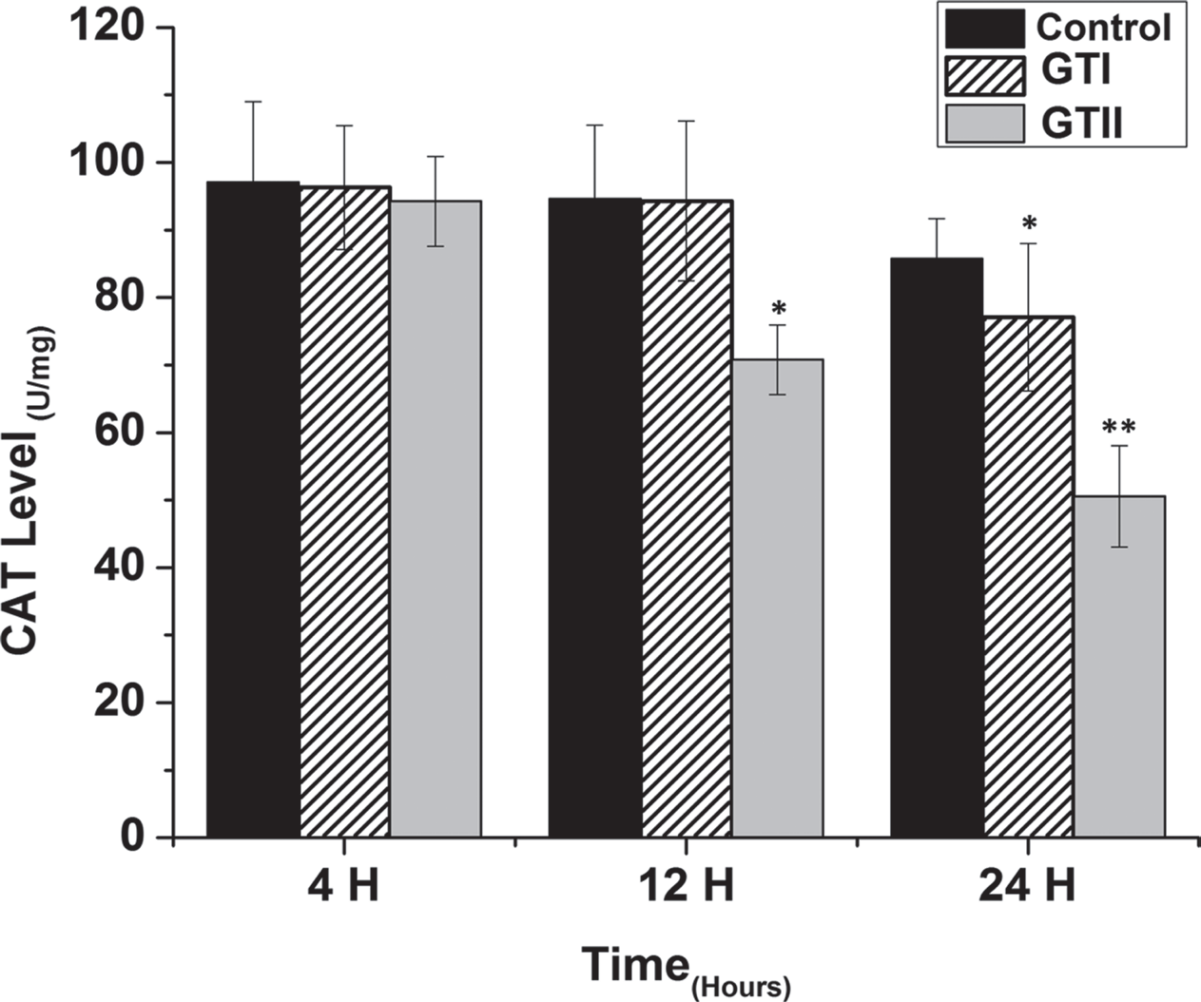
Effect of *P. zopfii* GTI and GTII on catalase (CAT) content The bMECs were challenged with GTI and GTII at 4 h, 12 h and 24 h. Results indicated the means ± SD of the three independent experiments. *p < 0.05, **p < 0.01.

**Figure 4:**
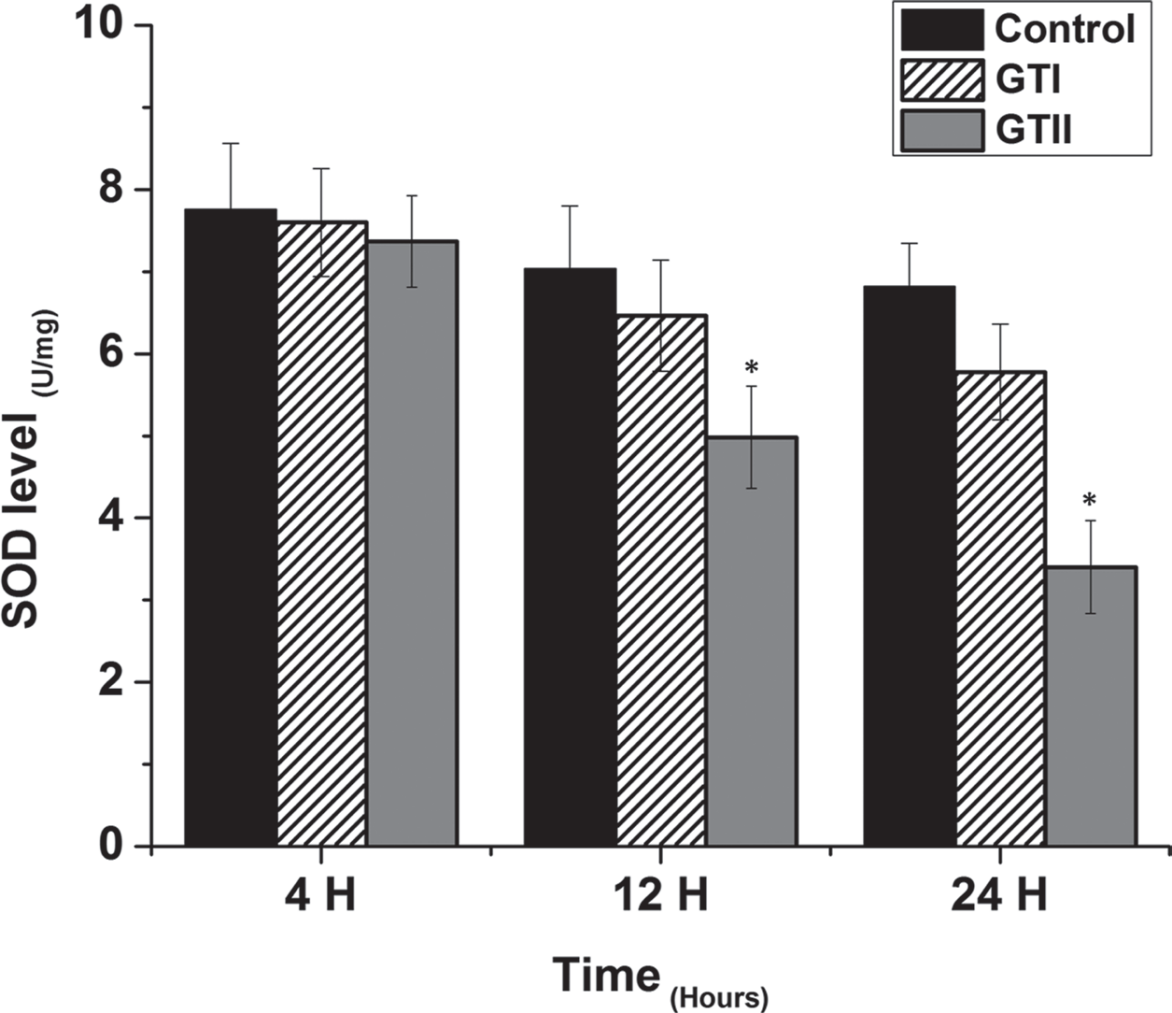
Effect of *P. zopfii* GTI and GTII on superoxide dismutase (SOD) level The bMECs were infected with GTI and GTII at 4 h, 12 h and 24 h. Data represent the means ± SD of the three independent experiments. *p < 0.05, **p < 0.01.

**Figure 5:**
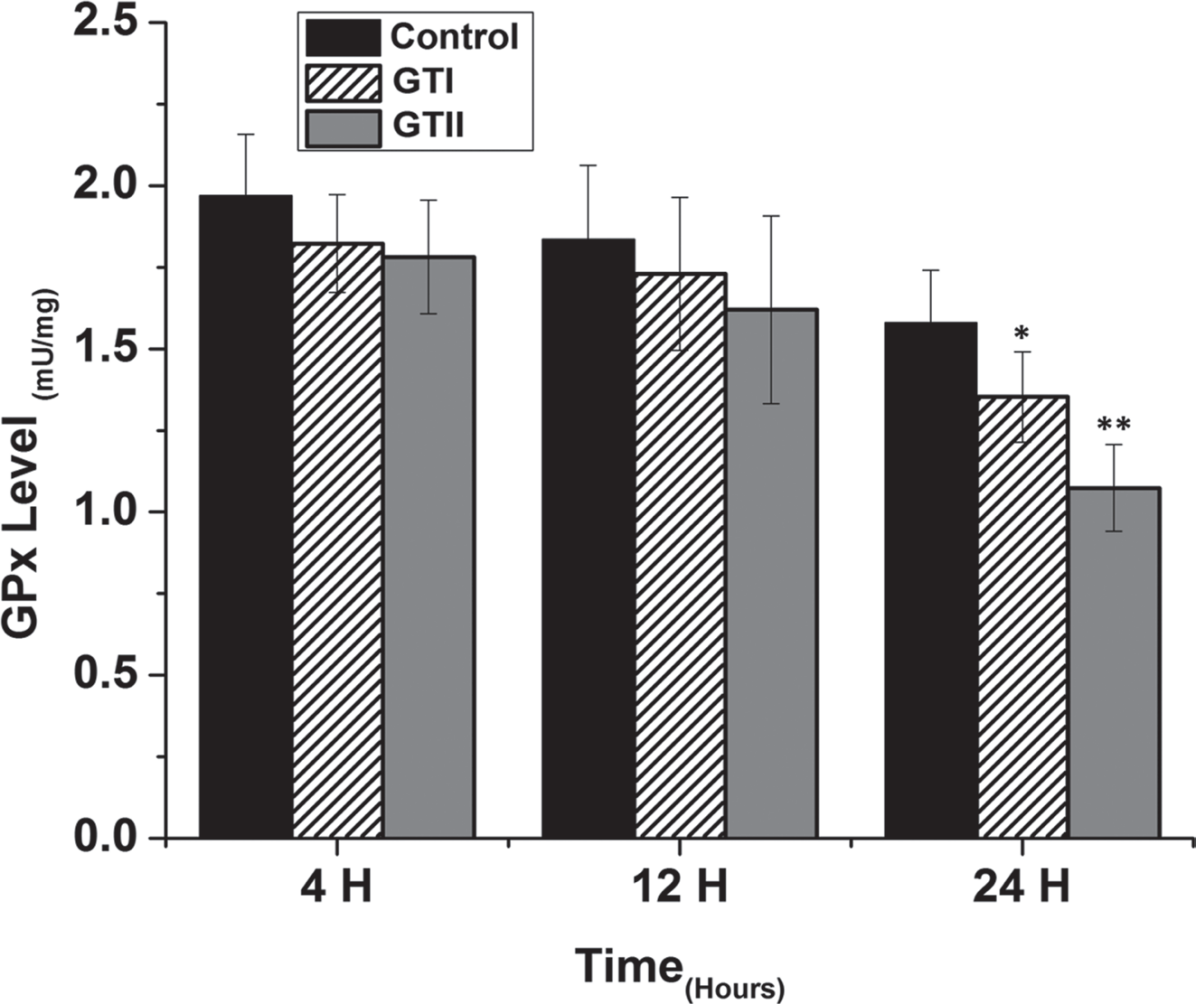
Effect of *P. zopfii* GTI and GTII on glutathione peroxidase (GPx) activity The bMECs were challenged with GTI and GTII at 4 h, 12 h and 24 h. Results indicated the means ± SD of the three independent experiments. *p < 0.05, **p < 0.01.

The results of antioxidant enzyme activities of GPx, SOD and CAT did not show any significant differences in both GTI and GTII treated and control group at 4 h post-infection (Figures 3, 4 and 5). At 12 hours, there was a significant decrease in the activities of CAT (70.77 ± 1.169, *p* < 0.05) and SOD (4.98 ± 0.62, *p* < 0.05) in GTII treated group in comparison with control group, while GTI infected group could not show any significance differences in various enzymes levels. At 24 hours of infection, there was a significant lower activities of CAT (50.55 ± 6.48, *p* < 0.01), SOD (3.402 ± 0.57, *p* < 0.05) and GPx (1.073 ± 0.13, *p* < 0.01) were observed in GTII bMECs. However, in case of GTI infected group, there were a slightly significant differences in CAT (77.07 ± 10.92, *p* < 0.05) and GPx (1.35 ± 0.038, *p* < 0.05) only at 24 h as compared to control group.

**Figure 6:**
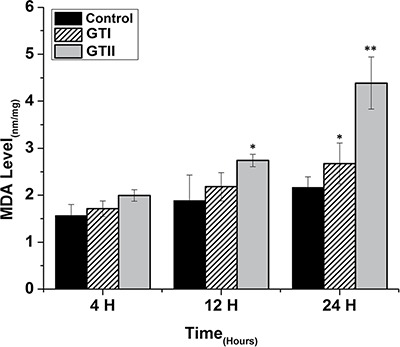
Effect of *P. zopfii* GTI and GTII on intracellular malondialdehyde (MDA) concentration The bMECs were infected with GTI and GTII at 4 h, 12 h and 24 h. Data represent the means ± SD of the three independent experiments. *p < 0.05, **p < 0.01.

In contrast, MDA content was significantly increased at 12 h (2.74 ± 0.073, *p* < 0.05) and 24 h (4.39 ± 0.35, *p* < 0.01) in GTII treated group compared with control group. While in case of GTI infection, there was significantly increased in MDA content only at 24 h (2.62 ± 0.34, *p* < 0.05) in comparison with control group, but the expression was lower than the GTII group as shown in Figure 6.

### *P. zopfii* triggered lactate dehydrogenase (LDH) release

**Figure 7:**
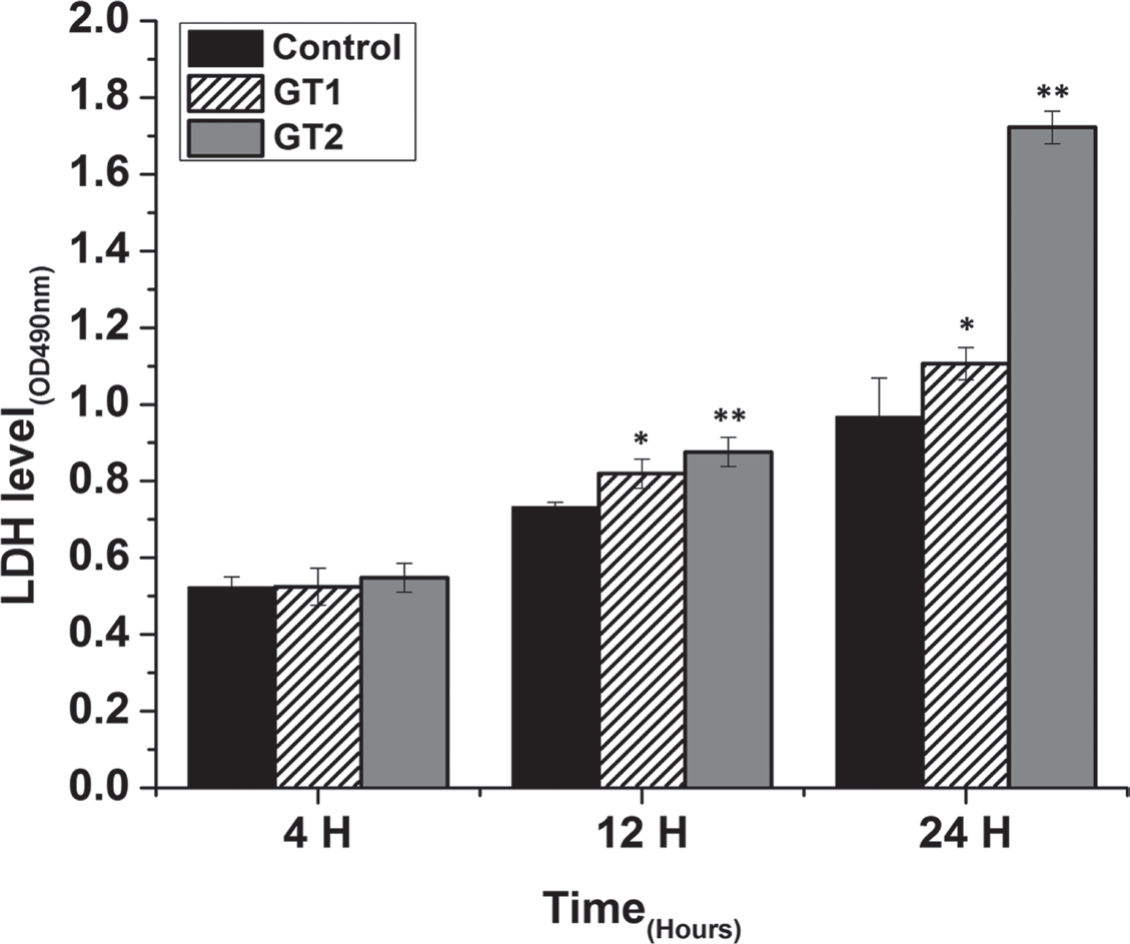
Effect of *P. zopfii* GTI and GTII on LDH activity in the medium of cultured bovine mammary epithelial cells Data indicated as mean ± SD of three independent experiments. *p < 0.05, **p < 0.01.

As shown in Figure 7, in case of GTII infection, there was significantly higher release of LDH activities at 12 h (0.876 ± 0.0382) and 24 h (1.72 ± 0.042) post-infection as compared with GTI infected and control groups. This indicated that GTII seriously damage the bMECs membranes with the passage of time. In case of GTI treated group, there was also significant increase (*p* < 0.05) of LDH level at 12 h (0.82 ± 0.038) and 24 h (1.106 ± 0.042) as compared to control group; however, this effect was lesser than GTII.

### *P. zopfii* stimulated reactive oxygen species (ROS)

**Figure 8:**
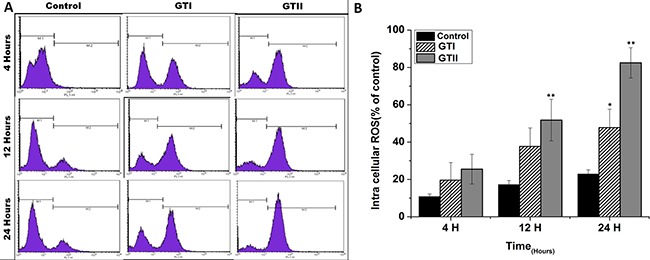
Comparison of ROS production in bMECs after infection of GTI and GTII by flow cytometry at 4 h, 12 h and 24 h Results showed the means ± SD of the three independent experiments. *p < 0.05, **p < 0.01.

**Figure 9:**
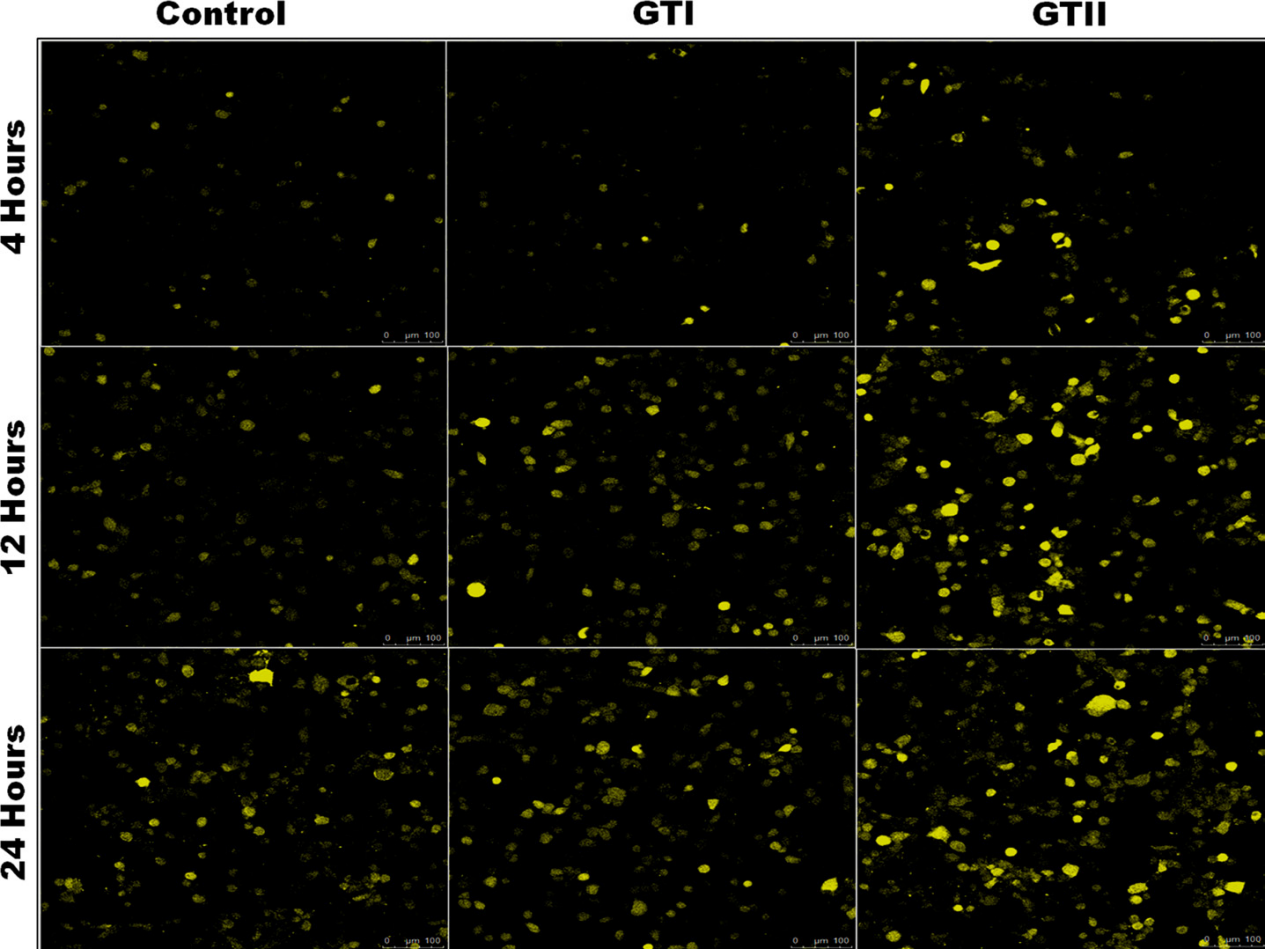
Effects of *P. zopfii* GTI and GTII on ROS production The bMECs challenged with GTI and GTII induced ROS production on time-dependent manner. ROS generation was observed using laser scanning confocal microscope.

ROS generation in bMECs after *P. zopfii* genotypes infection is shown in Figure 8. The production of ROS after GTII infection was significantly increased (50.29 ± 6.158 to 82.48 ± 8.078, *p* < 0.01) with the passage of time from 12 h to 24 h in comparison with GTI and control groups (Figure 8A, 8B). While in case of GTI infection, ROS production was increased only after 24 h of infection (47.72 ± 9.89, *p* < 0.05). Our findings of the confocal laser microscopy were also in compliance with the results of flow cytometry analysis (Figure 9).

## DISCUSSION

The mammary tissues during production have complex biochemical reactions and a vigorous metabolism. Bovine mastitis is associated with oxidative stress in which significant increase of reactive oxygen species (ROS), malondialdehyde (MDA) and nitric oxide (NO) and significant decrease of glutathione (GSH), that leads to inflammation and cell apoptosis [27, 28]. Therefore, the susceptibility of the mammary gland to immune dysfunction and inflammatory disorder decrease the health status of udder, milk production and quality of milk [29, 30]. *P. zopfii* GTII is a potential cause of bovine protothecal mastitis characterized as decrease in milk quantity and quality, and also associated with the abnormality of the effected quarters. Nevertheless, there is little published data about the pathogenesis of *P. zopfii* in bovine mastitis.

The present study was carried out to investigate the effect of *P. zopfii* on the oxidative stress indicators after *in vitro* infection of bMECs with *P. zopfii* GTI and GTII at 4 h, 12 h and 24 h. The antioxidant enzymes such as GPx, CAT and SOD could be used to examine the condition of different antioxidant defense mechanisms against free radicals [31]. The present data elaborated that the enzyme contents of GPx, CAT and SOD were significantly lower in *P. zopfii* GTII treated groups compared with control group, which indicated *P. zopfii* GTII induces oxidative cell damage in a time-dependent manner. The GTI infection only at 24 h significantly altered the CAT and GPx activities but the effect was less prominent than GTII. This may be attributed to the longer duration of GT1 infection which might cause lethal effects in the bMECs. However, previous studies conducted in various countries declared that GTII is the causative organism of bovine mastitis and GT1 is never isolated from cases of mastitis. The antioxidant enzymes like SOD, CAT and GPx can eliminate a variety of active oxygen free radicals. The over-expression of antioxidant has the ability to block the activation of nuclear factor kappa-light-chain-enhancer of activated B cells (NF-κB). Activated NF-κB can also decrease SOD and increase MDA levels in cells under oxidative stress [32]. The enzyme activities of GPx, SOD, CAT, and the level of MDA are vital indicators of oxidative damage from free radicals and lipid peroxidation [33]. Cells contain a variety of antioxidants that play an important role in protection against high release of ROS in tissues during mammary gland infection [34]. This study is the first report about *in vitro* antioxidant activity induced by *p. zopfii* in bMECs.

Apoptotic activity plays a pivotal role in the development of bovine mastitis. In the present study we performed the PI and annexin-V FITC staining which elaborated that GTII significantly induced apoptosis in bMECs. Additionally, hematoxylin and eosin staining demonstrated that treatment with *P. zopfii* GTII for 12 h and 24 h causes prominent disruption of bMECs. We also examined the effect of *P. zopfii*-induced ROS production in bMECs. This showed that *P. zopfii* GTII induced a significant increase in ROS generation after 12 h and 24 h of infection in bMECs. These results are suggestive of *P. zopfii* GTII induced bMECs apoptosis and oxidative stress via the imbalance of oxidant and antioxidant defenses as well as the production of intracellular ROS. Malhotra et al. reported that persistent oxidative damage and ROS generation can stimulate the protein misfolding and initiate apoptotic cascade [35], which is in agreement with our observation. As the anti-oxidative potential of the cells diminished, this results in increase production of ROS which is lethal for the survival of cells.

The findings of previous studies confirmed that the *P. zopfii* GTII is the etiological agent of bovine protothecal mastitis [8, 9, 26], this is in support of our study as *in vitro* infection of bMECs with GTII could significantly cause oxidative damages and apoptosis. However, in the current study the pathogenicity of *P. zopfii* was not confined to mammary gland, and immune responses to this pathogen *in vivo* may be much complex. This is needed to be further investigated by using *in vivo* model. The previous studies also confirmed the status of *P. zopfii* GTI as an environmental organism, without involvement in the pathology of mammary gland [8, 9, 26]. The severity and strength of the mammary gland immune response depends on the type of pathogen and its virulence [1]. The studies about the recognition of potential virulence factors in *P. zopfii* GTII are currently in progress and a recent immunoproteomic investigation in this context revealed that genotype specific antigen/epitopes could be identified as potential virulence factors in case of *P. zopfii* GTII [36]. In contrast, GTI may be deficient in such virulence factor, which might clarify its nonpathogenic behavior.

It is concluded that *P. zopfii* GTII induces oxidative damage and apoptosis. ROS generation and antioxidant activities in bMECs may play an important role in the pathogenesis of protothecal bovine mastitis. Further studies are needed to elaborate the specific mechanism involved in the oxidative stress induced by *P. zopfii* GTII.

## MATERIALS AND METHODS

### Protothecal strains used in the study

In the current study previously isolated strains of *P. zopfii* from dairy farms in Beijing were used, which were stored in our laboratory at College of Veterinary Medicine, China Agriculture University, Beijing [24–26]. The isolates were activated from stored stock by streaking on sabouraud dextrose agar (SDA, Difco™, Becton Dickison, Sparks, MD USA) and incubated at 37 °C for 48 h, then cultivated a single colony in sabouraud dextrose broth (SDB, Difco™, Becton Dickison, Sparks, MD USA) and incubated for 48 h. *P. zopfii* GTI and GTII were characterized by genotype specific primers [4]. Randomly selected three strains of each genotype (GTI and GTII) were used in following experiments, which were performed independently in triplicate.

### Cell culture

Bovine mammary epithelial cells (bMECs) lines MAC-T (Shanghai Jingma Biological Technology Co., Ltd. China) were used in this study and maintained as described in our previous study [23]. The bMECs were cultured in DME/F12 (HyClone, USA) medium along with Gibco® 10% fetal bovine serum (FBS; HyClone, USA), penicillin (100 U/mL; HyClone, USA) and streptomycin (100 U/mL; HyClone, USA) in cell culture plates (Corning, NY, USA).

### Infection of bMECs with *P. zopfii* genotypes

The bMECs were infected with *P. zopfii* GTI and GTII for 4 h, 12 h and 24 h; this was based on our previous work on cell viability and flow cytometry after protothecal infection for different time intervals (data not shown). BMECs were seeded on coverslips and incubated in 5% CO_2_ at 37°C. The logarithmic phase of GTI and GTII (5.0 × 10^5^ cells/well) were used in six-well plates.

### Annexin V–FITC and PI Staining

Apoptosis induced by *P. zopfii* in bMECs was interpreted by annexin V-FITC and PI staining method. The bMECs (1 × 10^5^ cells/mL) were cultured as mentioned above. These cells were infected with GTI and GTII for 4 h, 12 h and 24 h. After treatment, the coverslips were washed thrice with ice cold PBS and stained with FITC conjugated annexin-V mixture at room temperature (30 min), then bMECs were stained with PI (5 min) after twice washing with cold ice PBS, finally, stained bMECs were analyzed under confocal laser-scanning microscope (LEICA TCS SP5-Germany) with an excitation/emission 488/ 525 nm for FITC and excitation/emission 488/620 nm for PI. The proportion of the positive apoptotic bMECs were expressed as percentage of counted cells.

### Optical microscopy

The bMECs (1 × 10^5^ cells/mL) were seeded on glass coverslips as described above. The cells were infected with GTI and GTII (5 × 10^5^ CFU/mL) for 4 h, 12 h and 24 h. The cells on coverslips were immediately fixed in absolute ethanol and then processed with 90% and 70% alcohol followed by hematoxylin staining. Then cells were washed with tap water and treated with 70% alcohol for decolorization. After ammonia water treatment cells were counter stained with eosin. Finally, the cover slip was dipped in 95% alcohol and xylene for 30 sec. The cells were observed under an optical microscope (Olympus, Japan).

### Oxidative stress indicators analysis

The concentrations of antioxidant enzyme like catalase (CAT; U/mg), superoxide dismutase (SOD; U/mg), glutathione peroxidase (GPx; mU/mg) and oxidant enzyme like malondialdehyde (MDA; nmol/mg) in bMECs were detected using a colorimetric method, according to the manufacturer's instructions (Nanjing Jiancheng Bioengineering Institute, Nanjing, China). The readings were recorded by the STAT FAX 2100 automatic enzyme standard instrument (Awareness Technology, Inc. USA).

### Lactate dehydrogenase activity (LDH) assay

Lactate dehydrogenase (LDH) assay was used to assess the cytotoxic effect of GTI and GTII on bMECs. The bMECs (1 × 10^5^ cells/mL) were exposed to GTI and GTII at 4 h, 12 h and 24 h. The supernatants were collected after incubation, and LDH assay kit (Dojingdo Laboratories, Kumamoto, Japan) was used to estimate the LDH level by biochemistry analyzer.

### Assessment of reactive oxygen species (ROS)

The intracellular reactive oxygen species (ROS) in bMECs was evaluated using probe 2′, 7′-dichlorofluorescein diacetate (DCFH-DA). The bMECs (1 × 10^5^ cells/mL) were treated with GTI and GTII (5 × 10^5^ cells/mL) for 4 h, 12 h and 24 h. Infected cells were washed with PBS and treated with DCFH-DA (5 μmol/L) (20 min). After discarding the supernatant the treated cells were resuspended in PBS (two times). Fluorescence assays were measured and quantified by confocal laser-scanning microscope (LEICA TCS SP5-Germany) using excitation/emission 525/610 nm and also analyzed by flow cytometry (Beckman, Fullerton, California, USA) at excitation/emission 488/525 nm.

### Statistical analysis

The data were shown as mean value ± SD of the three independent experiments. The data were statistically evaluated by the one-way analysis of variance (ANOVA) followed by least significant difference (LSD) test for multiple comparisons, and post hoc test using SPSS 19.0 (SPSS, Inc., Chicago, IL, USA). Moreover, *p* < 0.05 and *p* < 0.01 represent the significance and highly significance differences.
